# Population-wide administration of single dose rifampicin for leprosy prevention in isolated communities: a three year follow-up feasibility study in Indonesia

**DOI:** 10.1186/s12879-018-3233-3

**Published:** 2018-07-11

**Authors:** Anuj Tiwari, Steaven Dandel, Rita Djupuri, Liesbeth Mieras, Jan Hendrik Richardus

**Affiliations:** 1 Department of Public Health, Erasmus MC, University Medical Center Rotterdam, P.O.Box 2040, 3000 CA Rotterdam, The Netherlands; 2 Netherlands Leprosy Relief, Jakarta, Indonesia; 3 Subdirectorate Tropical Disease of Leprosy and Yaws, Ministry of Health Indonesia, Jakarta, Indonesia; 4 Netherlands Leprosy Relief, Amsterdam, The Netherlands

**Keywords:** Leprosy prevention, Single dose rifampicin, Post exposure prophylaxis, Blanket approach, Feasibility

## Abstract

**Background:**

Indonesia ranking third in the world, regarding leprosy burden. Chemoprophylaxis is effective in reducing risk of developing leprosy among contacts. ‘Blanket approach’ is an operational strategy for leprosy post-exposure prophylaxis in which all members of an isolated community, high endemic for leprosy are screened and given a single dose of rifampicin (SDR) in the absence of signs and symptoms of leprosy. The objective is to assess the operational feasibility of a population-wide ‘blanket’ administration of SDR for leprosy prevention in isolated communities in a remote island.

**Methods:**

A prospective follow-up study was conducted in the year 2014, 2015 and 2016 in Lingat village of Selaru Island, Indonesia. During the first two visits, screening and SDR were provided, whereas only screening was conducted during the third visit. The demographic and clinical data were used for a descriptive analysis of the project coverage and leprosy epidemiology.

**Results:**

During the first two visits, 1671 persons (88%) were screened, 1499 (79%) received SDR, and 213 (11%) were excluded based on the exclusion criteria. During the first two visits, 43 (2.6%) cases were diagnosed with leprosy with a rate of 2263 per 100,000 population. The prevalence was highest in the age groups 15–24 and 25–49 years. Total, 14 (33%) cases had MB and 29 (67%) PB leprosy. Two cases (5%) had grade 2 disability. During the third visit, 10 new leprosy cases, with no grade 2 disability, were detected out of 1481 screened persons at the rate of 484 cases per 100,000 population (*n* = 2065 population in 2016). Among those screened during the third visit, there was a 50% reduction of leprosy among those who had previously received SDR compared to those who had not.

**Conclusion:**

With adequate planning and some additional investment, it is feasible to implement a blanket approach of chemoprophylaxis in a remote island of Indonesia, although effort needs to be made to cover as many people as possible in the first visit. Contingency plans need to be made to actively follow this village closely in the coming years and continue leprosy elimination efforts until no new cases are found any more.

**Electronic supplementary material:**

The online version of this article (10.1186/s12879-018-3233-3) contains supplementary material, which is available to authorized users.

## Background

Leprosy is an infectious disease caused by *Mycobacterium leprae* [1]. The disease burden has reduced in the last two decades, for which multidrug therapy (MDT) is largely credited [2, 3]. Unfortunately, this has not led to the incidence based global elimination of leprosy [4]. Currently, 136 countries report leprosy (210,758 new cases in 2015), indicating that transmission of *M. leprae* has not yet been interrupted [5]. Leprosy causes serious and irreversible nerve damage, which is a leading cause of disability among communicable diseases in developing countries [6]. Globally, 14,059 cases (0.25/100,000 population) were detected with grade 2 disability in 2015 [5].

Indonesia is an archipelagic island country with a population of 237.6 million [7]. Half of its population lives in rural areas (census 2010) and there are over 300 ethnic groups in the country [8]. The geographical and demographical diversity challenges health service delivery, especially in remote areas [9]. Indonesia contributes 8% (17,202 new cases in 2015) to the global leprosy burden, and ranks third after India and Brazil [5]. The majority (84%) of new cases have the multibacillary (MB) form of the disease, which is considered largely responsible for the transmission due to high bacterial load. Following intensified efforts by the National Leprosy Control Programme (NLCP) of the Indonesian government, the new case detection rate declined between 2005 and 2014 by 13.5% [10]. However, a recent modelling study predicted that the 2020 London Declaration target of leprosy elimination in terms of interruption of transmission will be difficult to achieve for Indonesia [11–13]. Stigmatization of leprosy is a severe problem in Indonesia and this also hampers efforts to find and treat leprosy patients [14–16].

Initially, leprosy control and later elimination efforts focused heavily on finding and treating patients [17], but attention is now shifting more towards prevention of the disease. The latest WHO Global Leprosy Strategy 2016–2020 has early case finding (through contact tracing and screening) high on the agenda [18]. Preventive interventions that are currently being considered are chemoprophylaxis and immunoprophylaxis [19]. Chemoprophylaxis or post-exposure prophylaxis (PEP) is often combined with other approaches such as contact tracing or ‘blanket’ approaches [20, 21]. ‘Blanket’ approach is a strategy in which all members in a given community are screened by trained health workers for leprosy and given post-exposure prophylaxis (PEP). This approach is potentially suitable for secluded high-endemic areas, such as small islands communities. The leprosy control programme of Indonesia is introducing new prevention methods. It is the first country in Southeast Asia to pilot PEP with a single dose of rifampicin (SDR) within the leprosy control programme for selective highly endemic districts. Indonesia gained valuable experience with contact tracing followed by SDR in Sampang district (East Java), Bima district (West Nusa Tenggara), and the blanket approach in Mumugu village, Papua. These experiences however, have not yet been documented scientifically and disseminated for wider learning. A single but important study from Indonesia concluded that the blanket approach with rifampicin is effective in reducing the incidence of leprosy [22]. It is very important to explore the feasibility and effectiveness of the blanket approach in detail, because the national programme plans to expand SDR to other highly endemic areas, many of them being remote islands.

The blanket approach in Lingat Village was implemented under the Leprosy Post Exposure Prophylaxis (LPEP) programme. The objective of this paper is to assess the operational feasibility of the population-wide ‘blanket’ administration of SDR for leprosy prevention in isolated communities, by documenting the implementation process and initial results of a pilot project in a remote island of Indonesia.

## Methods

### Intervention site

**Fig. 1:**
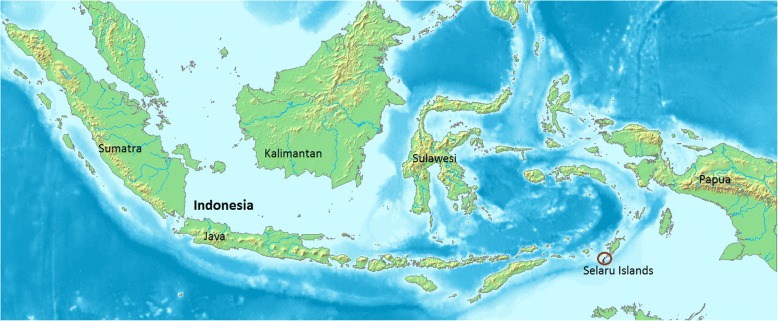
Location of Selaru Island in Indonesia (Source: Wikimedia Commons)

The blanket approach was conducted in Lingat village (population around 1900 in year 2014) in three visits in November 2014, November 2015 and November 2016. The village is situated on Selaru Island (Southeast Maluku West), which is a part of Tanimbar Islands in Maluku (Fig. 1).

The village can only be reached by boat and every year, due to poor weather conditions, the village is extremely hard to reach for several months. The NLCP received verbal information from the health workers of Tanimbar Islands that around 30 people were suspected of having leprosy. Photographs of the persons with patches were inspected by a leprosy expert. After initial confirmation, the government and non-governmental partners discussed the feasibility and agreed to organize a PEP blanket campaign. The selection criteria for the blanket approach are listed in Table 1.

**Table 1:** Feasibility criteria for a blanket approach campaign

Criteria	Desired Eligibility
Geographical location	Remote Island
The degree of isolation	More than 80% of the community members can be considered as contacts
Leprosy endemicity	CDR^a^ > 100/100,000
Sociocultural conditions	Openness for outsiders
Logistics and information	Mode of transportation, best time to visit as per weather conditions
Sustainability	The availability and training of health worker(s) to continue providing leprosy services

^a^*CDR* Case Detection Rate

### Study design

This is a three year prospective follow-up study, where implementation of the SDR intervention was completed during the first two visits with a year interval. During the third visit, the complete population of the island was screened again to establish the number of new cases arising since the implementation of the intervention during the first two visits. The campaign team first listed households, screened all available individuals and detected new leprosy cases, which were subsequently put on MDT. The remaining eligible population was offered SDR, after checking for the exclusion criteria, i.e. absence from the house, refusing consent, pregnant, suspect of having leprosy or diagnosed with leprosy, history of receiving rifampicin (for leprosy or any other disease) in the past two years, under the age of 2 years, and suspect of having tuberculosis. The aim was to reach at least 80% of the population, because a high coverage is desired to break the transmission chain by sufficiently reducing the bacterial load of *M. leprae* in the population. The target was not achieved during the first visit because many people were away from their houses to earn livelihood. Therefore, the first (baseline) visit was extended with a second visit in November 2015 to cover the absentees of the first year visit. Individuals who had received SDR during the first visit were not re-examined for leprosy during the second visit. During the study period, SDR was given only once to an individual.

### Study infrastructure

During the first visit, two teams were formed, each led by a medical doctor and supported by 2–3 leprosy health workers. The mode of transportation was speed motor boats, leaving from Saumlaki (the capital of the district) and sailing for 3 h to reach Lingat village. Each visit lasted 5–6 days. However, the number of teams was increased to five and six for the second and third visits. The details of the implementation process for all three visits are presented in Table 2.

**Table 2:** Standard steps followed while implementing the blanket approach

Steps	Details
1. Communication	Officially inform the local stakeholders about the blanket approach and schedule
2. Planning and logistics	- Training of the team - - Arrangements for transportation, medicines, supplies and stationary
3. Travel	A courtesy call to the village head
4. IEC	Community information on health, leprosy and purpose of visit
5. Screening	Door to door: - Line listing - Informed consent - Physical examination to diagnose leprosy - - Checking eligibility criteria
6. Medication/ Referral	- MDT to the newly identified cases - SDR to the eligible individuals (only in the first two visits) - - Biological sample collection for difficult to diagnose cases
7. Data collection	Simultaneously with step 5 and 6
8. Training of local health worker	- MDT treatment - Management of leprosy complications - - Follow-up of those who are absent or excluded to receive SDR

A standard set of data were collected on paper forms, and later entered electronically. An additional file shows this in more detail (see Additional file 1). The demographical and clinical data were used for the descriptive analysis and assessment of the project coverage and epidemiological situation.

### Statistical analysis

Data were subsequently entered into an excel-based electronic database during the visits. A unique identification number was generated for all personal records and matched to identify any previous contacts receiving SDR, but who later proved to be a leprosy case. Furthermore, contacts of the first and second visits were matched to check for overlap, because the second visit aimed to cover only those who missed the screening and SDR during the first visit. Data cleaning included assessment of missing values and logic checks for the related variables, e.g., age and dosage of rifampicin. Frequency distributions and subgroup analyses were performed for descriptive analysis. Groups were formed based on sex, age, type of leprosy and disability grade.

## Results

The officially registered population of the island in November 2014 was 1900, which increased to 2065 by November 2016. During the first two visits, 1743 (92%) of this estimated population were listed, 1671 (88%) screened, and 1499 (79%) received SDR. The screening covered 1241 people during the first visit, the remaining 430 people during the second visit, and 1481 people during the third visit (Table 3).

**Table 3:** The number of individuals listed (males and females), screened, found with leprosy, and given SDR

Visit	Sex	Listed	Screened	Leprosy	Prevalence (% screened)	SDR
Survey 1 (2014)	Male	621	589	17	2.9%	520
	Female	668	652	12	1.8%	584
	Sub total	1289	1241	29	2.3%	1104
Survey 2 (2015)	Male	232	214	10	4.7%	194
	Female	222	216	4	1.9%	201
	Sub total	454	430	14	3.3%	395
Total (population 1900 in 2014)		1743 (92%)	1671 (88%)	43	2.6%	1499 (79%)
					Incidence	
Survey 3 (2016)	Male	954	717	5	0.7%	NA
	Female	1000	764	5	0.7%	
Total (population 2065 in 2016)		1954 (95%)	1481 (72%)	10	0.7%	

The population was increased by the 3rd survey, which also included new individuals

In the first two visits, the male-female ratio of those screened and receiving SDR was nearly 1:1. In total, 43 (2.6%) persons were diagnosed with leprosy with the rate of 2263 per 100,000 population (*n* = 1900), of whom 16 (37%) were female. During the third visit, 10 persons were diagnosed with leprosy at the rate of 484 per 100,000 population (*n* = 2065), with equal distribution of cases by gender.

Table 4 shows the number of people screened and detected with leprosy by age group. In all the visits, children in the age 2–14 years formed the largest group, followed by adults aged 25–49 years, and adults above 50 years. In total 43 new leprosy cases (2.57%; 95% CI: 1.84–3.36%) were detected during the first two visits. Prevalence of leprosy was highest in those aged 15–24 years and 25–49 years (4.7 and 4.6%, respectively). During the third visit, 10 new leprosy cases (0.68%; 95% CI: 0.35–1.29%) were detected and treated. The incidence was again high in the age group of 25–49 years (70% cases).

**Table 4:** Number of cases screened and detected with leprosy in different age groups

	Survey 1 (2014)			Survey 2 (2015)			Survey 3 (2016)		
Age group in years	Screened	Leprosy	Prevalence %	Screened	Leprosy	Prevalence %	Screened	Leprosy	Incidence %
Under 2	66	0	0	5	0	0	48	0	0.0
02–14	514	8	1.6	164	1	0.6	469	3	0.6
15–24	100	3	3	49	4	8.2	142	0	0.0
25–49	297	12	4	119	7	5.9	449	7	1.6
≥ 50	264	6	2.3	93	2	2.2	373	0	0.0
Total	1241	29	2.3	430	14	3.3	1481	10	0.7

In the first two visits, 14 of the cases had MB leprosy (33%; 95% CI: 19–47%), and 29 PB leprosy (67%; 95% CI: 53–82%). Two people (5%; 95% CI: -2.18-6.18%) had grade 2 disability. In the third visit, 6 cases had MB leprosy (60%; 95% CI: 27–86%) out of 10 new cases, with no grade 2 disability (Table 5).

**Table 5:** PB-MB and G2D distribution according to sex in leprosy cases identified during three visits

Visit	Sex	Leprosy	MB	%	PB	%	G2D	%
Cases (2014)	Male	17	8	47	9	53	2	12
	Female	12	4	33	8	66	0	0
	Sub total	29	12	41	17	59	2	12
Cases (2015)	Male	10	0	0	10	100	0	0
	Female	4	2	50	2	50	0	0
	Sub total	14	2	14	12	86	0	0
Total		43	14	33	29	67	2	5
Cases (2016)	Male	5	3	60	2	40	0	0
	Female	5	3	60	2	40	0	0
	Sub total	10	6	60	4	40	0	0
Grand Total		53	20	38	33	62	2	4

During the first two visits, SDR was administered to 1499 persons. Out of the total number screened who had no signs or symptoms of leprosy, 213 were excluded on the basis of the exclusion criteria (Table 6). Being absent and under 2 years of age were the most common reasons for exclusion. During the third visit, 1481 people were screened, of whom 1071 had received SDR during the earlier visits and 410 had not because of exclusion criteria or absence. The number of new cases among the 1071 who had received SDR was 6 and the number of cases among the 410 who had not received SDR was 4 (Table 7). The odds ratio is 0.57 (95% CI: 0.16–2.03), indicating that the odds of having leprosy are lower in the exposed (SDR) than in the non-exposed (no SDR) group.

**Table 6:** Number and Reasons for Exclusion from SDR in the first two visits

Visit	Absent	Refused Consent	Pregnant	Suspect Leprosy	Rifampicin History	Under age 2	Suspect TB	Total
Contact (2014)	48	0	13	11	7	66	12	157
Contact (2015)	24	0	5	11	4	5	7	56
Total	72	0	18	22	11	71	19	213

**Table 7:** The details of new cases detected in the third visit

SDR history	SDR in visit	Age	Gender	Type
Received	1st	3	M	PB
Received	2nd	5	M	PB
Received	1st	25	M	MB
Received	1st	27	F	MB
Received	1st	44	M	PB
Received	1st	49	F	MB
Not received	NA	4	M	MB
Not received	NA	32	F	PB
Not received	NA	33	F	PB
Not received	NA	49	F	MB

*NA* Not applicable

## Discussion

The blanket approach in Lingat Village was implemented under the Leprosy Post Exposure Prophylaxis (LPEP) programme. The aim of LPEP is to assess impact and feasibility of contact tracing and administration of single dose of rifampicin (SDR) to asymptomatic contacts of leprosy cases in the six countries (India, Nepal, Myanmar, Sri Lanka, Tanzania, Indonesia) [23]. Indonesia however, is the only country where the blanket approach is applied. In the other countries SDR is provided to close contacts of leprosy patients only.

The objective of the blanket approach campaign was to survey the complete target population and provide all eligible individuals with SDR in one visit in a high endemic, isolated population. Because the desired coverage of more than 80% was not reached, it was decided to conduct a second visit in the next year to include those who were missed during the first visit. The inadequate coverage in the first visit was mainly due to lack of awareness in the population regarding leprosy and its consequences, although the officials and inhabitants were well informed prior to the visit (Table 2). The absentees were out to earn livelihood, because there were wage losses when staying at home in receiving the intervention. The skin examination can only be done in daylight, therefore late evening or early morning timings were not suitable, although this could increase the coverage. Certainly, the first visit increase awareness, and the second visit emphasized seriousness of the intervention, which helped to increase the coverage in the following visits. By the end of second visit, 92% were listed and 88% were screened. A third (follow-up) visit after a year was conducted to monitor the number of new cases arising in the population after the intervention during the two baseline visits. Among the people screened during the third visit, there was an apparent reduction of leprosy of around 50% among those who had previously received SDR compared to those who had not. However, a high rate of transmission is evident as 3 child cases (2–14 age group) were detected in the third visit, and 2 of them had SDR in the previous visits. Studies on effectiveness have been done before, but not always with clear conclusions, due to methodological shortcomings. In 1988, a non-controlled trial with SDR 25 mg/kg dose was implemented in the Southern Marquesas Islands [24–26]. The intervention achieved 98.7% coverage (2715 received SDR out of 2786 inhabitants), and additionally covered 3144 South Marquesans living elsewhere in French Polynesia. As a result, new 5 cases were detected in the next 10 years among treated population, which was significantly less than the 17 expected cases in a hypothetical situation of unchanged transmission rate. In comparison to the Polynesian population that did not receive the intervention, chemoprophylaxis was found to have an additional protective effect of 35–40%. In 1990, Pacific islands implemented chemoprophylaxis in the Federated States of Micronesia, Kiribati and the Republic of the Marshall Islands [27]. The screening covered 70% of the population for two consecutive years, including chemoprophylaxis (both years) of rifampicin-ofloxacin-minocycline (ROM) to adults and rifampicin only to children under 15 years of age [28]. By 1999 a substantial reduction in case detection was observed, but it could not be established that this was due to intervention [27].

In the year 2000, five high endemic islands in Indonesia piloted chemoprophylaxis with a defined control group [22] with 600 mg rifampicin for adults and 300 mg for children (6–14 years old) with approximately 3.5 months between doses. Two types of chemoprophylactic intervention strategies (blanket approach and contact tracing SDR) were compared with a control group (no chemoprophylaxis). In contact tracing SDR, prophylaxis was given to eligible contacts of all known and newly found leprosy patients only, unlike the blanket approach. The population cohort of 3965 persons was actively screened before the intervention and subsequently once a year for three years. The yearly incidence rate in the control group was 39/10,000; the cumulative incidence after three years was significantly lower in the blanket group. No difference was found between the contact tracing SDR and the control groups. This study showed that population-based prophylaxis was associated with a reduced leprosy incidence in the first three years after implementation. Subsequently the COLEP trial in Bangladesh, in which SDR was given to contacts of leprosy patients, showed an overall reduction in the incidence of leprosy in the first two years of 57% [22]. The initial protective effect was maintained, but no difference in incidence was seen between the placebo and rifampicin groups beyond two years [22, 29, 30].

Based on the preceding studies, it can be expected that the provision of chemoprophylaxis in a blanket approach to a well-defined highly endemic population will help reduce the transmission of *M. leprae* in that population. Apart from overall reduction of leprosy cases in the coming years, we do expect a possible relative increase in MB cases because SDR is more effective in reducing the PB cases due to lower bacterial load than MB. The increase in MB cases can also be due to previously missed early MB cases as it is often difficult to diagnose such cases through screening. One can expect that potential MB and PB cases that are early in the incubation period may respond well to SDR, but not those MB cases which are already advanced in their clinical stage. There are however, several important remaining questions regarding implementation aspects of such approach, and on the intensity and duration of active follow-up. It cannot be expected that leprosy will disappear by itself after a one-off intervention, as the experience of the Federated States of Micronesia and the Marshall Islands sadly demonstrated. The current study in Lingat village showed that the campaign could not be completed in just one visit. In order to optimize the effect of such campaign, an effort should be made to reach as much people in a single first round or to leave limited time between the first and second round, to avoid unidentified leprosy patients continuing to spread *M. leprae* in their surroundings. A one-year intercept is already quite long, and it would be preferable to conduct the second round within 6 months.

## Conclusion

The study in Lingat village shows that the blanket approach is in principal operationally feasible in terms of staff and time investment. It could likely be implemented in similar locations and sociocultural settings. The population preparation (prior information on timelines, methods and objectives of survey) is crucial in attaining the desired coverage. The people generally showed no resistance to the intervention and mostly accepted the SDR without refusal of consent. Most individuals excluded from SDR had genuine contraindications for receiving SDR. The intervention achieved a good balance in reaching males and females. The number of child cases was high (21%), indicating active transmission of *M. leprae* in the community. We recommend a close monitoring of children in the future, especially the under 2 years group, which was excluded from receiving SDR in the campaign. Furthermore, the intervention covered a relatively large group of children, probably because this group stays at home during the day, compared to other household members who are often out working.

So far, SDR appeared to show protective effect against leprosy during the third visit. We recommend to conduct a fourth visit in Lingat village after one year to re-screen the full population, so that the effectiveness of the SDR can be further established. There is no evidence yet regarding the number of rounds required to control or eliminate leprosy is such setting, and the desired time interval between rounds. Because we expect to find more leprosy cases in future, contingency plans need to be made to actively follow this village closely in the coming years and continue leprosy elimination efforts until no new cases are found any more.

## Additional file

Additional file 1: Type of data collected during the visits of blanket campaign. Type of data collected during the visits of blanket campaign. (DOCX 13 kb)

## Abbreviations

G2D: Grade II Disability
LPEP: Leprosy Post Exposure Prophylaxis
MB: Multibacillary
MDT: Multidrug therapy
NLCP: National Leprosy Control Programme
PB: Paucibacillary
PEP: Post-exposure prophylaxis
ROM: Rifampicin-Ofloxacin-Minocycline
SDR: Single dose of rifampicin
