# Supplementary material for: Population-wide administration of single dose rifampicin for leprosy prevention in isolated communities: a three year follow-up feasibility study in Indonesia
Source: BMC Infect Dis. 2018 Jul 11;18:324. doi: 10.1186/s12879-018-3233-3 (PMC6042242; doi:10.1186/s12879-018-3233-3)
Supplement: Supplementary file 1 — Type of data collected during the visits of blanket campaign. Type of data collected during the visits of blanket campaign. (DOCX 13 kb) [file 12879_2018_3233_MOESM1_ESM.docx]

**Additional file 1**

Type of data collected during the visits of blanket campaign

| **Activity** | **Outcome** | **Data collected** |
| --- | --- | --- |
| Screening | Leprosy positive  population | Registration number |
|  |  | Date of diagnosis |
|  |  | Age, sex |
|  |  | Disability grading (Grade 0, 1, and 2) |
|  |  | Type of leprosy (PB-MB) |
|  |  | Mode of detection |
|  |  | Exclusion criteria: refused consent, absent, no contacts |
|  | Remaining  Population | Registration number |
|  |  | Date of screening |
|  |  | Age, gender, sex |
|  |  | Education |
|  |  | Exclusion criteria (Table 7) |
|  |  | Rifampicin swallowed – dosage received |
